# Population Pharmacokinetic Analysis Proves Superiority of Continuous Infusion in PK/PD Target Attainment with Oxacillin in Staphylococcal Infections

**DOI:** 10.3390/antibiotics11121736

**Published:** 2022-12-01

**Authors:** Irena Murínová, Martin Švidrnoch, Tomáš Gucký, Jan Hlaváč, Pavel Michálek, Ondřej Slanař, Martin Šíma

**Affiliations:** 1 Department of Clinical Pharmacy, Military University Hospital Prague, 16902 Prague, Czech Republic; 2 Department of Applied Pharmacy, Faculty of Pharmacy, Masaryk University, 60177 Brno, Czech Republic; 3 Laboratory of Pharmacology and Toxicology, AGEL Laboratories, 74101 Novy Jicin, Czech Republic; 4 Department of Pharmacology, First Faculty of Medicine, Charles University and General University Hospital in Prague, 12800 Prague, Czech Republic; 5 Department of Anaesthesia and Intensive Care, First Faculty of Medicine, Charles University and General University Hospital in Prague, 12808 Prague, Czech Republic

**Keywords:** antibiotics, nonlinear mixed-effects modeling, glomerular filtration rate, dosing regimen, oxacillin, Monte Carlo simulations

## Abstract

Considering its very short elimination half-life, the approved oxacillin dosage might not be sufficient to maintain the pharmacokinetic/pharmacodynamics (PK/PD) target of time-dependent antibiotics. This study aimed to describe the population pharmacokinetics of oxacillin and to explore the probability of PK/PD target attainment by using various dosing regimens with oxacillin in staphylococcal infections. Both total and unbound oxacillin plasma concentrations retrieved as a part of routine therapeutic drug-monitoring practice were analyzed using nonlinear mixed-effects modeling. Monte Carlo simulations were used to generate the theoretical distribution of unbound oxacillin plasma concentration–time profiles at various dosage regimens. Data from 24 patients treated with oxacillin for staphylococcal infection have been included into the analysis. The volume of distribution of oxacillin in the population was 11.2 L, while the elimination rate constant baseline of 0.73 h^−1^ increased by 0.3 h^−1^ with each 1 mL/s/1.73 m^2^ of the estimated glomerular filtration rate (eGFR). The median value of oxacillin binding to plasma proteins was 86%. The superiority of continuous infusion in achieving target PK/PD values was demonstrated and dosing according to eGFR was proposed. Daily oxacillin doses of 9.5 g, 11 g, or 12.5 g administered by continuous infusion have been shown to be optimal for achieving target PK/PD values in patients with moderate, mild, or normal renal function, respectively.

## 1. Introduction

Oxacillin is a narrow-spectrum semisynthetic penicillin that belongs to the beta-lactam antibiotics used to treat infections caused by Gram-positive cocci. The isoxazole group, which is attached to the beta 6 position on the penicillin core, ensures penicillinase resistance [1]. This is the reason why it is used in clinical practice, especially for moderate-to-severe penicillin-resistant staphylococcal infections. It is mainly used for the treatment of infections of the skin and soft tissues, osteomyelitis, and endocarditis. It can also be used for the treatment of CNS, urinary, or respiratory infections [1,2]. Oxacillin replaced its predecessor methicillin and offers a better safety profile, especially limiting the risk of interstitial nephritis. Methicillin and oxacillin share similar pharmacokinetic/pharmacodynamic (PK/PD) properties. Only a year after the introduction of methicillin into practice, methicillin-resistant Staphylococcus aureus was discovered and its occurrence in the hospital environment is quite common today [3]. Its weighted mean population prevalence in the European economic area was 16.7% in 2020 but varies significantly with a clear north to south/east gradient [4].

After the administration of oxacillin (nowadays only parenterally), it quickly distributes into most tissues and fluids of the body, including cerebrospinal fluid and bones, and crosses the placenta. It has a low volume of distribution (Vd), which is approximately equivalent to extracellular fluid, and has a high level of protein binding (~90%) [1]. A part of the oxacillin dose is metabolized into active 5-hydroxymethyl and inactive penicilloic acid derivatives in the liver [5]. Oxacillin, same as its metabolites, is excreted primarily in the urine via tubular secretion and glomerular filtration; only a minor part is eliminated by bile or maybe in breast milk. The elimination half-life is approximately 0.5 h and could be prolonged in patients with renal impairment or by concomitant use of probenecid due to competitive inhibition of renal tubular secretion [1,2].

The approved dosage of oxacillin for common infections is 0.25–1 g every 4 or 6 h, administered intravenously or intramuscularly. For severe infections the dose can be escalated up to 12 g/day [2,6].

The effect of beta-lactam antibiotics is time-dependent. The time when unbound oxacillin plasma concentrations are maintained above the minimal inhibitory concentration (fT > MIC) is considered as the most appropriate PK/PD target. The lower-limit threshold is set to fT > MIC = 40–70% (% of time between dosages) and the optimum is fT > MIC = 100%, but some studies have argued for an even more forceful target of fT > 4 × MIC = 100% [7,8].

Given the very short elimination half-life of oxacillin and its time-dependent antibiotic effect, it seems that approved dosages administered via standard infusion might not be sufficient to achieve this PK/PD target. The issue of achieving the optimal PK/PD target has already been addressed by other studies with the halogenated oxacillin derivatives cloxacillin and flucloxacillin, and they agreed that extended (3 h) or continuous infusion is more efficient than the standard 0.5 h infusion [9,10].

This study aimed to describe the pharmacokinetics of oxacillin using a population approach and to explore the probability of PK/PD target attainment using various dosing regimens with oxacillin in staphylococcal infections.

## 2. Results

A total of 24 patients (14 males, 10 females) were enrolled in the analysis. Their demographic and laboratory characteristics are summarized in Table 1. Patients received oxacillin to treat staphylococcal infections of the central nervous system (*n* = 7), sepsis (*n* = 3), orthopedic (*n* = 10), or other infections (*n* = 4) (e.g., bacteriuria, bacteremia, or endocarditis). Oxacillin doses ranged between 1 g and 3 g every 4 h and were administered via a 0.5 h intravenous infusion. Only one patient was given oxacillin via an extended 3 h infusion. Infection was caused by methicillin-susceptible *Staphylococcus aureus* in 21 cases, by *Staphylococcus hominis* in 2 cases, and by *Staphylococcus warneri* in one case. The median (IQR) value of MIC was 0.25 (0.25–0.5) mg/L (i.e., the most frequent MIC value was 0.25 mg/L).

A total of 32 oxacillin plasma concentrations were included in the PK analysis, with an average of 1.33 concentrations per patient. Twenty-four concentration points were taken as trough level, while eight samples were taken as peak level (sample collection after infusion completion). The median (IQR) value of oxacillin binding to plasma proteins was 86% (83–88%). Oxacillin binding to plasma proteins was not significantly associated with any of the covariates tested (total oxacillin plasma level, serum albumin, sex, age, body weight, height, BMI, and eGFR).

### 2.1. Population PK Analysis

Total plasma oxacillin concentration–time data were best fitted using a one-compartmental model with linear elimination kinetics. A proportional error model was the most accurate for residual and interpatient variability. The population model was parametrized using volume of distribution (Vd) and elimination rate constant (K_e_). The population PK estimates for the oxacillin final model are summarized in Table 2. Among the investigated variables, the most significant covariate was eGFR for oxacillin K_e_. The population Vd of oxacillin was 11.2 L, while K_e_ started at a baseline of 0.73 h^−1^ and increased by 0.3 h^−1^ with each 1 mL/s/1.73 m^2^ of eGFR.

The final equations describing the relationships between oxacillin PK parameters and their covariate are as follows:

Log(Vd) = log(Vd_pop) + η_Vd

Log(K_e_) = log(K_e__pop) + β_K_e__eGFR×eGFR + η_K_e_

where pop represents the typical value of the parameter, β represents the covariate effect on the parameter, and η represents a random effect variable.

**Figure 1:**
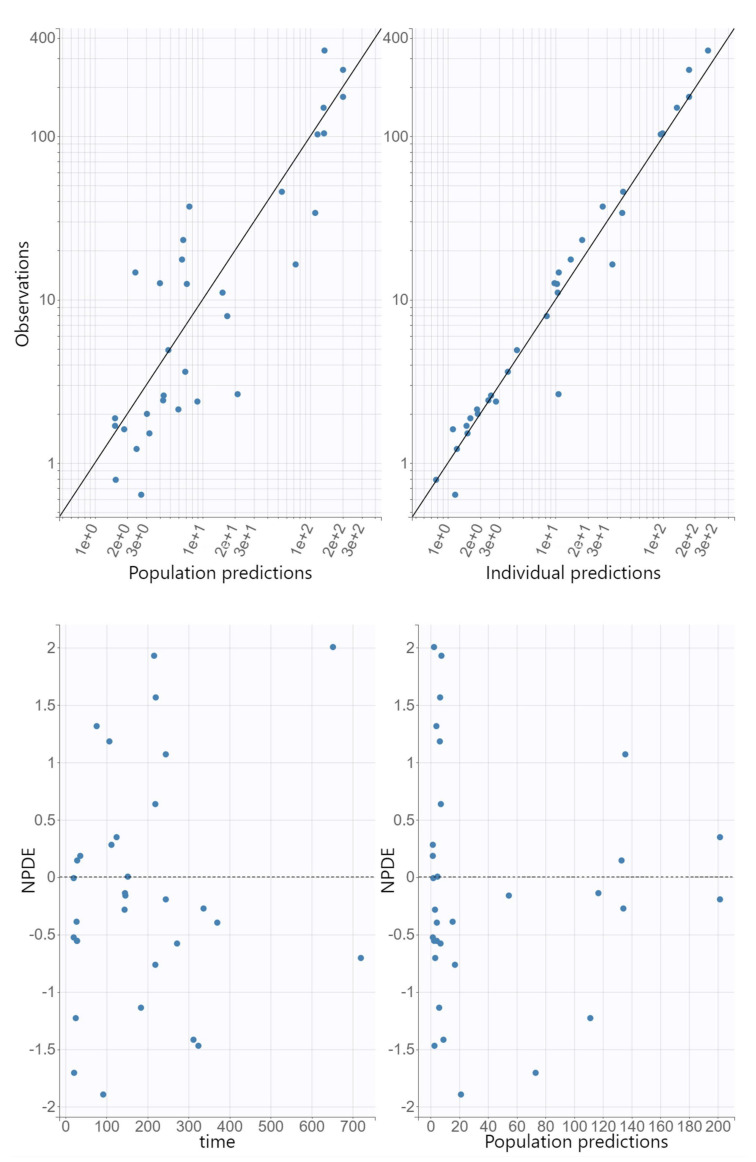
Population and individual predictions of oxacillin versus observed concentrations (log–log scale), and normalized prediction distribution errors (NPDE) versus time and population predictions.

**Figure 2:**
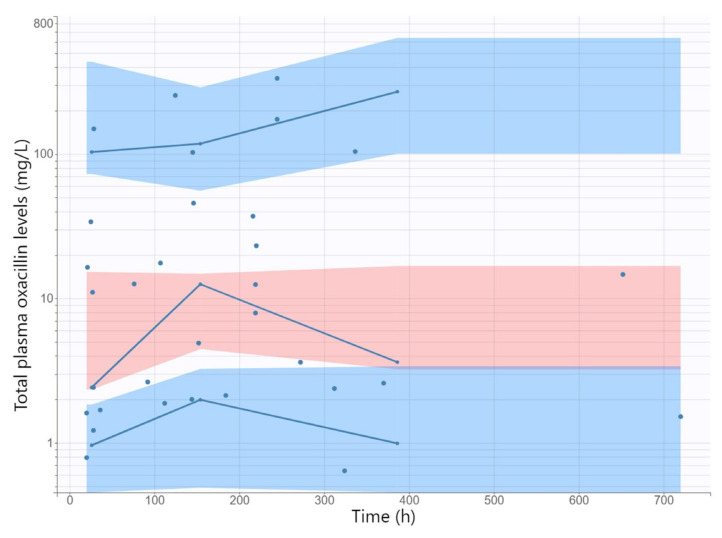
Semilogarithmically plotted visual predictive check and the observed data of the total plasma oxacillin concentrations in time for the final model. Solid lines represent the 10th, 50th, and 90th percentiles of the observed data. Shaded regions represent 90% confidence interval around the 10th (below blue region), 50th (pink region), and 90th (above blue region) percentiles of the simulated data.

The GOF plots for the final covariate model for oxacillin showed no substantial deviations (Figure 1). The R.S.E. values documented that the PK parameters in the population model for oxacillin were correctly estimated (Table 2). The VPC plot of the final oxacillin model showed that the predictions corresponded to the observed data, confirming the validity of the model to predict the PK data (Figure 2).

### 2.2. Monte Carlo Simulations

**Figure 3:**
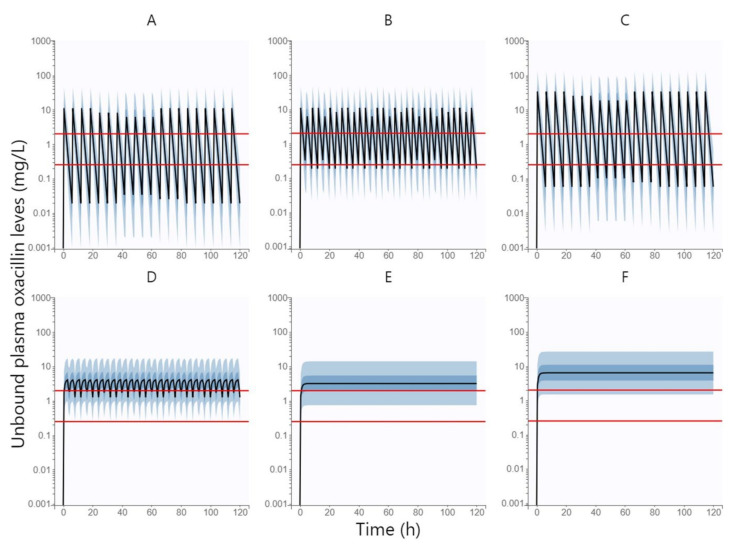
Simulated unbound plasma concentration profiles of oxacillin over time following intravenous administration of oxacillin at a dose of 1 g every 6 h by 0.5 h infusion (**A**), 1 g every 4 h by 0.5 h infusion (**B**), 3 g every 6 h by 0.5 h infusion (**C**), 1 g every 4 h by 3 h infusion (**D**), 6 g daily by continuous infusion (**E**), and 12 g daily by continuous infusion (**F**). The curve represents the median, and the four bands represent percentiles (5–27.5%, 27.5–50%, 50–72.5%, and 72.5–95%) of the 90% distribution of simulated concentrations. Red lines represent two different MIC values (0.25 and 2 mg/L).

Figure 3 shows the simulated unbound plasma concentration profiles of oxacillin over time (500 replicates of all subjects in the dataset) following intravenous administration of oxacillin at a dose of 1 g every 6 h by 0.5 h infusion, 1 g every 4 h by 0.5 h infusion, 3 g every 6 h by 0.5 h infusion, 1 g every 4 h by 3 h infusion, 6 g daily by continuous infusion, and 12 g daily by continuous infusion.

Table 3 summarized the PTA values of all above-mentioned oxacillin dosing regimens at various MIC values (0.25 and 2 mg/L) and various PK/PD targets (fT > MIC = 100% and fT > MIC = 50%). The dosing regimen was considered to be successful when ≥ 90% of patients reached the PK/PD target. The standard 0.5 h infusion was sufficient neither by shortening the dosing interval to 4 h nor by increasing the dose size to 3 g. Extended 3 h infusion can only be used at low MIC values (e.g., 0.25 mg/L), whereas continuous infusion has been shown to be effective in achieving PK/PD outcomes even when targeting higher MIC values. However, if we want to cover the PK/PD target up to the EUCAST epidemiological cut-off value for oxacillin of 2 mg/L, the dose needs to be increased to 12 g per day, administered via continuous infusion.

In a simulation of oxacillin administered by continuous infusion at a dose graded according to eGFR (daily dose of 9.5 g, 11 g, and 12.5 g in patients with moderate renal impairment, mild renal impairment, and normal renal function, respectively), the PTA was 90.18%; therefore, this dosing can be considered successful in achieving the optimal PK/PD target of fT > MIC = 100% with the EUCAST epidemiological cut-off value for oxacillin of 2 mg/L using the lowest possible doses.

## 3. Discussion

Oxacillin has been used for many years as one of the first-choice drugs in the treatment of staphylococcal infections [11]. Nevertheless, considering its very short elimination half-life and time-dependent antibiotic effect, the approved dosage administered via standard 0.5 h infusion might not be sufficient to maintain the PK/PD target. This widespread practice may then be the cause of frequent staphylococcal resistance [12]. To prevent the development of this unfavorable trend, antibiotic dosing needs to be optimized to achieve the PK/PD target in the majority of the population. Therefore, therapeutic drug monitoring of oxacillin has been implemented into clinical routines in our hospital and optimized oxacillin dosing has been proposed based on a one-year follow-up population analysis of the acquired data.

Based on our analysis, the population volume of the distribution of oxacillin was 11.2 L, which, at median a body weight of 84 kg, corresponds to a body-weight-normalized Vd of 0.13 L/kg. However, the patients in our study were overweight (median BMI of 28 kg/m^2^) and if we converted their weight to a normal BMI value of 22 kg/m^2^, then the body-weight-normalized Vd comes out to 0.17 L/kg. This value is consistent with the volume of extracellular water into which the beta-lactam antibiotics were distributed [13]. The only significant covariate of oxacillin pharmacokinetics in our population model was eGFR for oxacillin K_e_, which started at a baseline of 0.73 h^−1^ and increased by 0.3 h^−1^ with each 1 mL/s/1.73 m^2^ of eGFR. In patients with an eGFR of 1.5 mL/s/1.73 m^2^ (normal renal function), the oxacillin K_e_ was therefore 1.18 h^−1^, which corresponds to the elimination half-life of 0.6 h. This value is fully consistent with that reported by the summary of product characteristics [2] and it is also the reason for the difficulty in achieving the PK/PD target at standard dosing.

While experimental data reported the antibiotic effects of beta-lactams at fT > MIC = 40–70%, clinical studies showed that a higher threshold of fT > MIC = 100 should be considered [7,8]. In our study, we explored the probability of target attainment for two pharmacological outcomes: fT > MIC = 100% was defined as an optimal PK/PD target, while fT > MIC = 50% was a minimal PK/PD target. However, considering the severity of some staphylococcal infections (e.g., endocarditis, sepsis, osteomyelitis, or pneumonia), the risk of developing resistance, and that therapeutic beta-lactam monitoring is usually not routinely implemented, we consider a higher PK/PD target to be more appropriate for the optimal dosage proposal. Both PK/PD target attainments were also explored for two different MIC values, i.e., 0.25 mg/L as the most frequent MIC value in our study and 2 mg/L as the EUCAST epidemiological cut-off value for oxacillin [14]. Again, we recommend assuming a higher MIC value in clinical routines if we do not have exactly measured strain-specific MIC values.

It should also be kept in mind that only the unbound fraction of the drug can exert an antibiotic effect. Since oxacillin is highly bound to plasma proteins, its unbound fraction should be measured, or at least estimated, from the total level using a correction factor. In our study, oxacillin binding to plasma proteins ranged from 74 to 97%, with a median value of 86%. This value is only slightly lower than that reported for healthy volunteers (89–94%) [2]. Since oxacillin binds primarily to albumin, an association between the unbound fraction and serum albumin level would be expected. However, we did not observe any significant relation. This can be explained by the fact that there were no patients with severe hypoalbuminemia in our study in whom the effect of increasing the unbound fraction of oxacillin would be most pronounced.

Monte Carlo simulations based on the oxacillin population pharmacokinetic model confirmed our assumption that the approved dosage administered via standard 0.5 h infusion is not sufficient to reach the PK/PD target. To achieve a higher PTA, dose intensification is needed, which can be done by several approaches—increasing the dose sizes, shortening the dosing interval, or extending the duration of the infusion. Increasing the dose sizes leads to only a small increase in PTA, which is logical for an antibiotic with a time-dependent effect. A shortening of the dosing interval would certainly be appropriate, but a dosing interval of less than 4 h would already be highly impractical for clinical routines. Therefore, extending the duration of the infusion is the most appropriate approach. As we can see in Table 3, an extended 3 h infusion is sufficient to cover the PK/PD target only at lower MIC values (0.25 mg/L); therefore, continuous infusion seems to be optimal. Another advantage of continuous infusion is that it shows the same PTA rate for both lower (fT > MIC = 50%) and higher (fT > MIC = 100%) PK/PD targets, i.e., if we reach the lower PK/PD target, we spontaneously reach the higher one. Thus, our findings confirmed our hypothesis that oxacillin should ideally be administered via continuous infusion, which is also consistent with the dosing recommendations for flucloxacillin and cloxacillin from the other studies that have addressed this issue [9,10]. However, if we want to cover the PK/PD target up to the EUCAST epidemiological cut-off value for oxacillin of 2 mg/L in the whole population, the daily dose administered via continuous infusion needs to be increased up to 12 g per day. To cover this PK/PD target with the lowest possible daily doses, we proposed a dosage graded by eGFR as a covariate of oxacillin elimination. This intention is best met by daily doses of 9.5 g, 11 g, and 12.5 g in patients with moderate renal impairment, mild renal impairment, and normal renal function, respectively.

We acknowledge several limitations of our study. Firstly, we evaluated only the achievement of the PK/PD target, not the real clinical outcomes. Secondly, since our analysis was based on TDM data from clinical routines, the majority of samples were taken as a trough concentration, and thus the distribution phase in our population model may not be exhaustively described. On the other hand, diagnostics of our model did not show any inaccuracies throughout the monitoring time course. Moreover, for oxacillin as a time-dependent antibiotic, the elimination phase is crucial for achieving the PK/PD target. Therefore, our dosing proposal should not be biased by the sampling limitation. Finally, since patients with severe renal impairment or with augmented renal function were not present in our study cohort, we are unable to suggest dosing for these extreme cases.

## 4. Materials and Methods

### 4.1. Study Design

A retrospective observational pharmacokinetic study was conducted in adults (age ≥ 18 years) treated with oxacillin intravenous infusion for staphylococcal infection admitted to mixed wards of the Military University Hospital in Prague from June 2021 to June 2022. Patients were included in the study if they had at least one measurement of oxacillin plasma level during treatment. Exclusion criteria were extracorporeal life support and renal replacement therapy. The study was approved by the local ethics committee of the Military University Hospital in Prague under the registration number 108/17–98/2022, and followed the principles established by the Declaration of Helsinki. Due to the retrospective nature of this study, which involved only analysis of routine clinical data, study-specific informed consent for was waived. The collection and processing of anonymized data is in the public interest.

### 4.2. Data Retrieval

The clinical records of all included patients were reviewed to collect information on age, sex, height, and body weight, as well as serum creatinine, urea, and albumin levels. Body mass index (BMI) was computed as the ratio of body weight (kg) to the square of height (m). For each patient, the glomerular filtration rate (eGFR) was calculated according to the chronic kidney disease epidemiology collaboration formula. Oxacillin dosing regimens, including administration times and infusion rates, were recorded. Both total and unbound oxacillin serum concentrations were determined as a routine part of the therapeutic drug-monitoring procedure. Sampling times were also collected. Minimal inhibitory concentration (MIC) value of oxacillin for isolated staphylococcal strain was also recorded in each patient.

### 4.3. Bioanalytical Assay

#### 4.3.1. Chemicals and Reagents

Acetonitrile (ACN), methanol, and water (all of them HPLC-MS grade) were supplied by Chem-Lab NV (Zedelgem, Belgium). Ammonium acetate (≥99.9%), formic acid (≥98%), and oxacillin sodium salt monohydrate were purchased from Sigma-Aldrich (St. Louis, MO, USA). Amoxicillin-d4 (≥98%, internal standard) was purchased from Toronto Research Chemicals (Toronto, Canada). Human sera from more than 10 volunteers of different sexes and ages were pooled and used as matrix. The drug-free status was verified by measuring a blank sample of the pooled serum. Calibration samples of oxacillin were prepared by direct spiking of the verified serum to final concentrations of 1, 5, 10, 50, and 100 mg/L.

#### 4.3.2. Instrumentation

The LC-MS/MS analyses were performed using a Shimadzu LC-MS system Nexera X2 LC-30AD coupled with a LCMS-8060 triple quadrupole mass spectrometer. All separations were carried out on an Kinetex^®^ C18 column (2.6 µm, 3 × 50 mm; Phenomenex, Torrance, CA, USA) thermostated to 40 °C. The LC system consisted of two binary pumps, a solvent rack equipped with a degasser, thermostated autosampler, and a column oven. The separation conditions used were as follows: sample injection volume 1 µL; mobile phases (MF): (A) consisted of water/ACN (9:1, *v/v*) containing 0.1% (*v*/*v*) of formic acid and 10 mM ammonium acetate and (B) consisted of water containing 0.1% (*v*/*v*) of formic acid and 10 mM ammonium acetate. The separation was conducted in an isocratic mode with a flow rate of 0.2 mL/min (60% MF A and 40% MF B). The LC-MS/MS system was operated with an electrospray ionization probe in a positive mode. The MS/MS detection was performed using a multiple-reaction monitoring mode (MRM) with the following MRM transitions: *m/z* 402.2→144.0 (quantifier) and 402.2→186.0 (qualifier) for oxacillin and *m/z* 368.2→227.2 for amoxicillin-d4.

#### 4.3.3. Sample Preparation and Quantification

A very high fraction of oxacillin is bound to plasma proteins; thus, an ultrafiltration step was applied to isolate the free fraction of oxacillin. An amount of 500 μL of serum aliquot was transferred to the centrifugal filter (Centrifree^®^ PL Regenerated Cellulose, 30 kDa; Sigma-Aldrich, St. Louis, MO, USA) and centrifuged at 1000 rcf for 15 min. To 30 uL of sample (serum and ultrafiltrate) were added 10 μL of internal standard solution (amoxicillin-d4) and 60 μL of ACN. The solution was vortexed and centrifuged at 20,000× *g* for 5 min.

Supernatants were transferred to plastic inserts, placed in vials, and analyzed. A five-point calibration method was used for quantification of the clinical samples.

### 4.4. Statistics

Descriptive parameter medians and interquartile ranges (IQRs) were calculated using MS Excel 2013 (Microsoft Corporation, Redmond, WA, USA). Mann–Whitney U-test and linear regression model were used to evaluate the relationships between oxacillin binding to plasma proteins and categorical and continuous variables, respectively. GraphPad Prism software version 8.2.1 (GraphPad Inc., La Jolla, CA, USA) was used for all comparisons, and *p*-levels less than 0.05 were considered statistically significant.

### 4.5. Population PK Analysis

Oxacillin plasma concentrations against time were analyzed using nonlinear mixed-effects modeling. The model parameters were assumed to be log-normally distributed and were estimated by maximum likelihood using the stochastic approximation expectation maximization algorithm within the Monolix Suite software version 2021R1 (Lixoft SAS, Antony, France). The model was built in three steps.

(1) *Base model*

One- and two-compartmental models with first-order or Michaelis–Menten elimination were tested for the structural model. Log-normally distributed interindividual variability terms with estimated variance were tested on each PK parameter. Proportional, additive, and combination error models were tested for the residual error model. The most appropriate model was selected based on the minimum objective function value (OFV), adequacy of the goodness-of-fit (GOF) plots, and low relative standard errors (R.S.E.) of the estimated PK parameters.

(2) *Covariate model*

Age, bodyweight, height, body mass index, and serum creatinine, urea, and albumin levels, as well as eGFR were tested as continuous covariates (characteristics predictive of interindividual variability), while sex and the reason for oxacillin treatment were tested as categorical covariates. Preliminary graphical assessment and univariate associations using Pearson’s correlation test for the effects of covariates on PK estimates was performed. Covariates with *p* < 0.05 were considered for the covariate model. A stepwise covariate modeling procedure was then performed. For model selection, a decrease in OFV of >3.84 points between nested models (*p* < 0.05) was considered statistically significant, assuming a χ^2^-distribution. Other criteria for model selection were reasonably low R.S.E. values of the structural model parameter estimates, physiological plausibility of the parameter values obtained, and absence of bias in the GOF plots.

(3) *Model evaluation*

The adequacy of the model was assessed using GOF graphs. Observed values were plotted against individual and population prediction values. The normalized prediction distribution errors (NPDE) were plotted against time to assess randomness around the line of unity. The visual predictive check (VPC) was conducted to assess the predictability of the final model. For this purpose, 1000 replicate values of the original dataset were simulated using the final model parameter estimates, and the simulated distribution was compared with the distribution from the observations. From all replicates, 90% prediction intervals for the 10th, 50th, and 90th percentile of the simulations were computed and graphically presented.

### 4.6. Monte Carlo Simulations

Monte Carlo simulations (500 replicates of all the individuals in dataset) based on a final population PK model of oxacillin were performed to generate theoretical distribution of unbound oxacillin plasma concentration profiles over time using Simulx version 2021 (Lixoft SAS, Antony, France). The following oxacillin dosing regimens were simulated: 1 g every 6 h by 0.5 h infusion, 1 g every 4 h by 0.5 h infusion, 3 g every 6 h by 0.5 h infusion, 1 g every 4 h by 3 h infusion, 6 g daily by continuous infusion, and 12 g daily by continuous infusion.

As an optimal PK/PD target was considered if unbound oxacillin plasma concentrations are maintained above the minimal inhibitory concentration for the entire dosing interval (fT > MIC = 100%), but we also tested the achievement of fT > MIC = 50% as a minimal PK/PD target in beta-lactam antibiotics. Probability of target attainment (PTA) at steady-state was calculated for all dosage regimens and different MIC values–0.25 mg/L (as the most frequent MIC value in our study) and 2 mg/L (as an EUCAST epidemiological cut-off value for oxacillin) [14]. Chi-square test was used for evaluation of differences in PTA between various dosing regimens.

In order to propose an optimal dosing regimen that would cover the PK/PD target with the EUCAST epidemiological cut-off value for oxacillin of 2 mg/L using the lowest possible dose, administration of oxacillin continuous infusion in dose scaled by eGFR as the main covariate of oxacillin PK was subsequently simulated. Patients with moderate renal impairment (eGFR = 0.5–1.0 mL/s/1.73 m^2^) received oxacillin daily dose of 9.5 g, patients with mild renal impairment (eGFR = 1.0–1.5 mL/s/1.73 m^2^) received daily dose of 11 g, and patients with normal renal function (eGFR ˃ 1.5 mL/s/1.73 m^2^) received daily dose of 12.5 g.

## 5. Conclusions

We described oxacillin population pharmacokinetics in patients with staphylococcal infection. The only significant covariate was eGFR for oxacillin clearance. The median value of oxacillin binding to plasma proteins was 86%. We proved the superiority of continuous infusion in the attainment of the PK/PD target and proposed an eGFR-scaled dosing. If we want to target the optimal PK/PD target of fT > MIC = 100%, with a EUCAST epidemiological cut-off value for oxacillin of 2 mg/L, the daily oxacillin doses of 9.5 g, 11 g, and 12.5 g should be administered via continuous infusion in patients with moderate renal impairment, mild renal impairment, and normal renal function, respectively. As this study only assessed the PK/PD target achievement, confirmatory trials exploring real clinical outcomes would be needed.

## Figures and Tables

**Table 1:** Demographic and laboratory characteristics of patients.

Characteristics	Median	IQR	Range
Age (years)	55	45–72	26–84
Body weight (kg)	84	74–96	57–145
Height (cm)	174	168–182	153–195
Body mass index (kg/m^2^)	28	23–33	20–39
Serum creatinine (μmol/L)	82	65–92	50–151
eGFR (mL/s/1.73 m^2^)	1.53	0.98–1.71	0.59–2.10
Serum urea (mmol/L)	4.5	3.3–5.6	1.8–11.8
Serum albumin (g/L)	32	26–37	22–44

eGFR is estimated glomerular filtration rate according to the CKD-EPI formula. IQR is interquartile range.

**Table 2:** Estimates of the final oxacillin population pharmacokinetic model.

Parameter	Estimate	R.S.E. (%)
*Fixed effects*		
Vd_pop (L)	11.2	30.4
K_e__pop (h^−1^)	0.73	26.1
β_K_e__eGFR (h^−1^ per each 1 mL/s/1.73 m^2^ of eGFR)	0.3	50.8
***Standard deviation of the random effects***		
Ω_Vd	0.7	22.5
Ω_K_e_	0.11	37.5
***Error model parameters***		
b	0.4	19.8

Vd is oxacillin volume of distribution, K_e_ is oxacillin elimination rate constant, and eGFR is estimated glomerular filtration rate.

**Table 3:** Probability of target attainment at steady-state after intravenous administration of oxacillin in various dosing regimens (1 g every 6 h via 0.5 h infusion, 1 g every 4 h via 0.5 h infusion, 3 g every 6 h via 0.5 h infusion, 1 g every 4 h via 3 h infusion, 6 g per day via continuous infusion, and 12 g per day via continuous infusion) at various MIC values (0.25 and 2 mg/L) and various PK/PD targets (fT > MIC = 100% and fT > MIC = 50%).

	Probability of Target Attainment (%)			
PK/PD Target	fT > MIC = 100%		fT > MIC = 50%	
MIC	0.25 mg/L	2 mg/L	0.25 mg/L	2 mg/L
1 g every 6 h (0.5 h infusion)	4.35	0.05	60.83	5.55
1 g every 4 h (0.5 h infusion)	41.52	2.60	60.25	27.69
3 g every 6 h (0.5 h infusion)	17.56	0.64	87.77	27.48
1 g every 4 h (3 h infusion)	95.30	32.23	99.93	72.40
6 g/day (continuous infusion)	99.82	69.95	99.82	69.95
12 g/day (continuous infusion)	99.97	90.72	99.97	90.72
*p*-Value	˂0.0001	˂0.0001	˂0.0001	˂0.0001

## Data Availability

The data that support the findings of this study are available from the corresponding author upon reasonable request.
